# Telepresence Robot System for People with Speech or Mobility Disabilities

**DOI:** 10.3390/s22228746

**Published:** 2022-11-12

**Authors:** Hebah ElGibreen, Ghada Al Ali, Rawan AlMegren, Reema AlEid, Samar AlQahtani

**Affiliations:** 1 Information Technology Department, College of Computer and Information Sciences, King Saud University, Riyadh 11451, Saudi Arabia; 2 Artificial Intelligent Center of Advanced Studies, King Saud University, Riyadh 145111, Saudi Arabia; 3 Center of Smart Robotics Research, College of Computer and Information Sciences, King Saud University, Riyadh 11543, Saudi Arabia

**Keywords:** telepresence, robotics, speech impairment, mobility impairment

## Abstract

Due to an increase in the number of disabled people around the world, inclusive solutions are becoming a priority. People with disabilities may encounter many problems and may not be able to easily participate in various activities due to physical barriers, which may sometimes cause them to be frustrated and embarrassed. Recently, the emerging telepresence robot technology has been proposed to enable people with disabilities to increase their presence by incorporating information and communications technology (ICT) into robotics platforms. Therefore, in this paper we conduct a comprehensive analysis using comparative and elicitation studies to understand the current state of mobile telepresence robot systems and to identify the gaps that must be filled. This paper further contributes to the literature by proposing a novel telepresence robot system that adapts text-to-speech (TTS) and ICT technologies with robotics for its use as an assistant. To the authors’ knowledge, the proposed system is the first MRP system that supports speech impairment and introduces emotion components into its communication function. It includes an operator site (mobile) and a remote site (robot) to allow users to control the robot from a distance and communicate with others in remote locations. It allows the user to physically interact with people and show certain emotions through the robot in remote locations, or it can accompany them to speak on their behalf. It can provide agency for both remote and in-class users through emoji-based communication and audio–video streaming with recording functionality. As shown at the end of this paper, the system was tested with 30 people, some of whom had mobility or speech disabilities, showing that the user acceptance score was above 95% and that people with disabilities liked to interact with other people using the proposed system. The users appreciated having the ability to control the robot from a distance and praised the capability to show their emotions through the robot emoji motions and to control the audio–video streaming. From this study, we conclude that the proposed telepresence system could be an asset to people with speech and mobility disabilities and could help them feel physically present in various places.

## 1. Introduction

With the annual increase in the number of people with disabilities [1], supporting disabled people with inclusive solutions has become a worldwide challenge. People with special needs face difficulties when communicating with other people due to either logistics or physical challenges. They cannot easily participate in various activities such as education programs or work meetings due to their physical conditions. Speech impairment is a disability where people have difficulty with oral communication, such as stuttering, speech disorders, or dysphonia of different stages and categories [2], while some people cannot speak at all. A mobility disability is another challenge that makes it difficult for a disabled person to freely move in some spaces. Thus, communication and socialization can be difficult, which makes these individuals frustrated and embarrassed in some cases, and many of them avoid social events or face-to-face meetings.

Although the current teleconferencing tools can be used for such problems, they are limited in different aspects [3]. The participants cannot see or hear everyone in remote locations, and it can be frustrating and difficult to speak if the person has a speech disability. People with speech impairments say that they are often affected and feel sad when others try to complete or finish a sentence on their behalf [4], which is one of the reasons why they do not participate in conversations and feel shy when talking to others. Therefore, a more advanced technology known as robotics telepresence technology has been introduced.

Telepresence [5,6] is the experience of being present in a real-world location. Robotics telepresence, also known as telerobotics, is a subfield of telepresence that aims to increase people’s presence by incorporating ICT technologies into robotics platforms [7]. In particular, “*A telepresence robot is a computer, tablet, or smartphone-controlled robot, which allows those who engage with the robot to view and hear the robot’s operator while the operator can view what the robot is “looking” at and hear sound at the robot’s location*” [8]. In short, the telepresence robot makes the distant person closer and enables him or her to do what he or she wants from his or her place without being physically present. Telepresence robots are also known as virtual presence robots or remote presence robots. A telepresence robot can make a person’s daily life easier; for example, a dynamic robot can allow its controller to conduct a meeting without being physically present [8]. Although telepresence robots can sometimes be expensive, they can save users other expenses such as travel costs or fees, which the user may need to pay when using other tools [9].

Robots were introduced to the health care field decades ago, and although a huge portion of the elderly population could not keep up with technology, there was a positive psychological impact from interacting with a robot [10]. In particular, socially assistive robotics (SAR) have been successfully used with patients with Alzheimer’s for memory training and cognitive stimulation [11]. Different robots have been introduced into the market for SAR. Martın et al. [12] summarized the literature results for using SAR with elderly individuals and found the following: (1) elderly individuals commonly liked the robots; (2) how robots look can influence their acceptance; (3) robots could improve humor and decrease loneliness; (4) robots could improve communication and memories of the past; (5) the use of robots could reduce dementia severity in some cases.

Telepresence robotics technology has recently been realized as a tool that can overcome traditional teleconferencing challenges and allow the user to control the robot and physically interact with people in remote locations or accompany the user to speak on their behalf. A study conducted by Tsui et al. [13] confirmed that people spoke to telepresence robots similarly to how they spoke to another person. Another study by Thompson and Chaivisit [14] suggested that telepresence robots had the potential to provide agency to both remote and in-class users. One important type of telepresence robot technology is known in the literature as mobile robot telepresence (MRP). MRP enables social contact via video conferencing with the ability to move and orient the system to other areas [15]. The primary objective of MRP systems is to facilitate social interactions between humans from a distance. The system consists of both a physical robot (sensors and actuators) and the interface to guide the robot. The design of MRP systems varies according to the manner where they are used and their application. MRP systems are used to enhance interactions with different communities, since they can be applied in several fields, such as health care and office environments. However, as discussed in the literature, many research gaps must be overcome.

Furthermore, as emphasized by Eiben [16], the current state of the art is limited when it comes to real robot applications; most studies rely on simulations, while the use of systems in the real world are scarce. Park et.al. [17] also confirmed that social robots have great potential to provide emotional, behavioral, and social support to people with diverse characteristics and needs. However, evaluating the acceptance and efficacy of these robots requires the deployment of real-world systems with real users.

Therefore, this paper contributes to the literature on MRP by first conducting an intensive study to understand the current state of the MRP literature. This study includes two main components: (1) a comparative study to understand the competitive systems available in the literature; (2) an elicitation study to understand the current state of MRP users and identify the user-centered needs and requirements in our local community. Regarding the comparative study, it is possible to compare the current MRP systems and highlight the strengths and weaknesses of each system. This study demonstrates that the current literature on MRP lacks information on artificial intelligence (AI) components, user experiences and considerations of multilayer disabilities, social interactions from a distance, and the accessibility of the proposed system. Meanwhile, the elicitation study shows that the local community in Saudi Arabia has difficulty accessing MRP systems, although they are using other expensive technologies that are less efficient than MRP. Thus, there is a need for more systems that are locally accessible, which motivated the elicitation requirements to build the proposed solution.

Second, this paper contributes to the literature by developing a new MRP system that introduces AI technologies by adding text-to-speech (TTS) capability to the robot. To the authors’ knowledge, the proposed system is the first MRP system that supports speech impairments and introduces emotion components to its communication function. This system will include an Android mobile application that can be downloaded on the user’s mobile phone and a robot application that allows the user to control the robot from a distance and interact with other users at remote locations. This system introduces AI components and adapts information and communication technologies (ICT) with robotics for use as an assistant or even a telepresence avatar of its owner. Moreover, the user can command the robot in parallel with the submitted text speech to show certain emotions, which can improve social interactions and communication with users at a distance. Other MRP functionalities are also introduced, such as navigation, video–audio streaming, and recording. The main purpose of this system is to help people with mobility and speech impairments interact with others, even if they are not physically present. Therefore, the proposed MRP system contributes to the literature from different aspects, as follows:

- We develop a novel framework that allows users to efficiently navigate indoor/outdoor surroundings with simple interactions with the robot;
- We design an easy-to-use and portable mobile-based interface for people with disabilities to control the robot;
- We utilize an online AI-based speech-to-text module that allows people with a speech disability to talk through the robot;
- We develop an emoji control module for human interaction that allows the user to control how the robot reacts with a certain speech approach;
- We introduce a recording module to the robot to allow the user to track the robot’s daily activities.

Third, an experimental study is conducted with 30 possible users who test the proposed system. The goal of the evaluation study is to test the software architecture and the user capabilities for navigation, as well as the robot control and communication through the system. Moreover, participants’ reactions toward the possible adoption of the developed robot system in everyday activities are assessed, resulting in a score above 95%. This result indicates that the proposed system can be an asset to people with speech or mobility disabilities and can help them feel physically present in the targeted place.

This paper is organized as follows. First, the related work and comparative analysis results are discussed. Then, the requirement elicitation process is presented, and its results are explained. Then, the proposed MRP system is presented and the proposed robot framework and experimental results are discussed. Finally, the paper is concluded and future study directions are proposed.

## 2. Comparative Study

**Figure 1:**
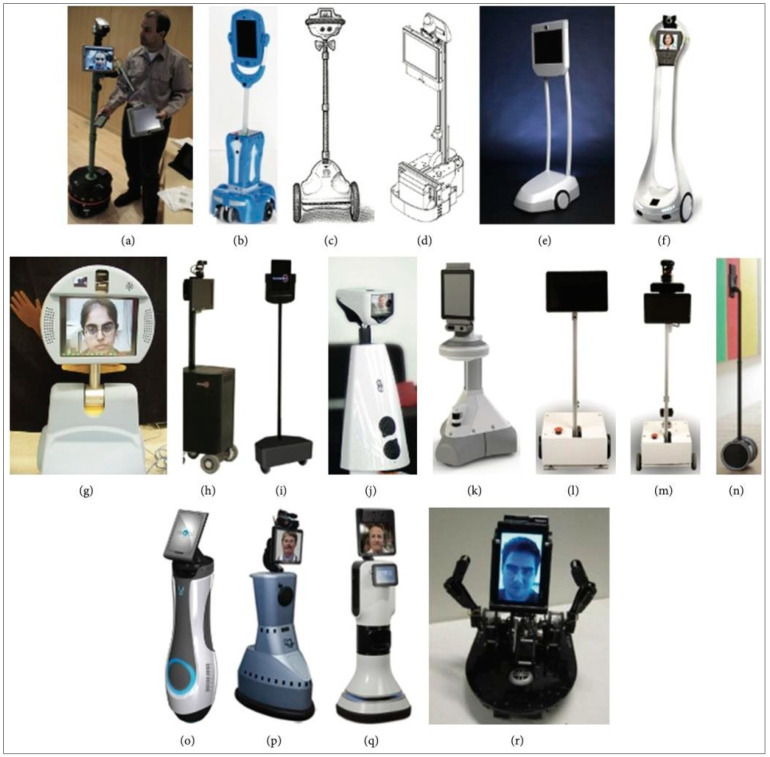
The most well-known MRP robots: (**a**) PRoP; (**b**) Giraff; (**c**) QB; (**d**) Texai; (**e**) Beam; (**f**) VGo; (**g**) PEBBLES; (**h**) MantaroBot Classic; (**i**) MantaroBotTeleMe; (**j**) Jazz Connect; (**k**) iRobot Ava; (**l**) 9th Sense Helo; (**m**) 9th Sense Telo; (**n**) Double; (**o**) mObi; (**p**) RP-7; (**q**) RP-VITA; (**r**) MeBot [18].

A study on social robotic telepresence systems [18] with a mobility focus to cover different types of MRP systems was conducted by Kristoffersson et al., as summarized in Figure 1. Each robot targets a different area of application, such as office environments, health care, care for elderly and aging people, school settings, and general MRP Systems. For example, a telepresence robot named the QB robot was used to allow employees to work remotely. It is possible to reduce the employees’ need to travel and consequently their travel expenses. The user can control the QB robot’s movement via a laptop keyboard. The main disadvantage of using the QB telepresence robot is that the user must have a laptop to control the robot. Thus, although there are many telepresence robotic systems, most of the described applications have their own challenges, and the evaluation results of these challenges can be considered in future MRP systems. The successful interaction and usability of the interfaces will provide a good communication experience for pilot users and local users, and they can provide added value for future MRP systems.

Newhart et al. [19] proposed a new mobile telepresence robot that helped students who could not attend school because of chronic diseases such as heart disease and cancer. This system allows a student who stays at home or is in hospital to control the telepresence robot to make him or her feel as if he or she were physically in school using the Wi-Fi connection between the student and robot. The telepresence robot has a screen to show the student’s face. In addition, it enables the student to talk to and hear his or her classmates. The robot provides a feature that emulates raising a hand in class by using flashing lights. The main drawbacks of this robot are the complexity of controlling the robot with a computer mouse and its inaccessibility because a laptop must be used with the robot.

In [20], a mobile telepresence robot named the Robovie-R3 was used to help children learn a new language from a teacher in a foreign country. The robot was used to overcome the problems of video conferencing, where children often freeze when talking in different languages and teachers find it difficult to engage with students from foreign countries. The Robovie-R3 robot was used on the teacher’s side, and the child controlled the robot in a remote place. The robot allowed the child to navigate (forward, backward, left, and right) and open and close the robot’s right hand by using a glove with a motion sensor (ZMP IMU-Z). However, this setup required extra equipment to move the telepresence robot, and a laptop must be used to operate the robot, which further increased the inflexibility.

Park and Howard [21] attached a Kinect depth sensor to a Pioneer 3AT robot and used it with a haptic feedback channel as a mobile telepresence robot for visually impaired people. The telepresence robot provided a 3D perception of the remote environment and nonvisually transferred information to allow users with visual impairment to interact with the environment. Different sensors were used to visualize the environment, such as a camera and an RGB-D sensor. Moreover, a haptic interface was used to control the robot in two ways: (1) via keyboards using the arrow keys; (2) by clicking on the position in the interface to which he or she wanted the robot to move. The built robot is very beneficial to visually impaired people because it also provides an auditory feedback channel, in addition to 3D perception. The auditory feedback channel is used to describe the status of the robotic system movement, such as when moving forward, left, right, backward, and stopping, in addition to its color and distance. However, its inflexibility is a concern because the user needs special hardware for the haptic feedback channel.

Another mobile telepresence robot called the Pi robot was developed by the Center for Advanced Study in the Behavioral Sciences (CASBS) in Stanford, California [3]. The main purpose of developing this robot was to serve people with limited physical mobility. The robot was tested by academics in the university who suffered from spinal cord injuries and could not walk. The Pi robot was improved in stages depending on the user feedback, and the final version included a pan-and-tilt camera and navigation features (for obstacle detection and avoidance). It also included a web-based user interface, a Nexus tablet for two-way audio–video chats via Skype, and an extra speaker to increase the audio output. Although the Pi robot has been successfully made into a telepresence robot, the authors stated that the robot was delicate and not available to the public.

In [22], a telepresence robot named the TeleMe robot was used to help students and teachers who could not attend school. The user can view live activities and meetings while controlling the movement of the robot so that it can move around the room. The teleconferencing ability of the robot runs through Skype, and an additional application (called MantaroBot) must be installed to control the robot. The user can remotely control the robot via a control application that communicates with the TeleMe robot whenever a Skype connection to the TeleMe is established. Thus, the user must have two applications simultaneously open to control the robot and view its stream.

Beams [23] is a telepresence robot that has been used for academic conferences. This robot is approximately five feet tall and has a display on top of it and two cameras. One camera looks outwards to provide a first-person perspective to the remote attendee, while the other camera faces down toward the robot’s base to facilitate navigation. The robot’s base is equipped with wheels that enable it to move forward, backward, and sideways. A web interface allows remote users to connect to a Beam robot. With the mirror feature in the bottom right corner of the web interface, the remote user can see how he or she appears on the robot’s display. Remote users can drive the Beam robot using a keyboard, mouse, or Xbox controller, which can sometimes be challenging.

The Double Robotics Company designed a robot called Double [24], which is a self-driving video-conferencing robot used for enhanced remote learning and working. During the COVID-19 pandemic, the University of Nevada, Las Vegas (UNLV) used a Double robot to help nursing and medical students improve their skills and attend medical courses. The main features of the double robot are self-balancing and the ability to control the height of the robot. It has other sensors such as two cameras: the iPad camera and a downward camera to connect the robot to the charging dock. Many fields use the Double robot, such as in business, higher education, K-12 education, and telemedicine. However, users may face difficulties controlling the robot because it provides click-to-derive functionality, which may limit the type of movement available to the user.

In [25], a robotic assistive platform called Marvin was proposed to provide indoor assistance to users. The proposed system allowed the robot to monitor users, assist the user at night, and introduce remote presence and connectivity through other tools such as Skype and WhatsApp. The targeted users for the proposed system were mostly elderly individuals who need assistance in their daily activities. From the experiment, a qualitative demonstration was conducted to measure the platform capability. Although the system proposed is autonomous and can be used as a social assistant to elders, the communication and telepresence functionality does not provide distance control and navigation. Thus, it is used for indoor setups and is only managed by its owner.

From the discussed literature, important features of MRP systems can be summarized, as shown in Table 1. From the table, these MRP systems are either developed for research purposes and not accessible to the public or only available through a commercial company that costs more to use. Even if the system is conducted for research, the robot used is mostly commercialized. Although the literature on MRP has covered different types of users, those with speech impairments have not been targeted. TTS functionality is very important for helping people with a speech impairment. However, no MRP robots so far have been endowed with this function. In addition, the use of emojis and emotion interaction, which is a good method to express the user’s feelings, has not been achieved. Another important feature missing from the literature is the ability to record videos, although it can be a useful function for the user when they want to view previous meetings. Finally, regarding the controlling device used by the pilot user to control the robot, all of these robots need extra devices to control the robot, and the users must press buttons for each motion, which can be difficult for people with a multilayer disability or elderly individuals. This setup can be a burden to the user, since he or she must carry around an extra tool or PC to control the telepresence robot.

Compared to previous studies, the proposed system in this paper introduces the following key factors to enhance MRP applications for people with disabilities:

- **Flexible Mobile System:** The proposed MRP system is complemented with a mobile-based application that guarantees for the user the ability to install and control the robot from any place at any time.
- **Diverse Communication:** The proposed system offers different means of communication to fit diverse types of users, including video–audio streaming, TTS communication, and emoji interaction.
- **Ease of Control:** An adaptable interface is designed to offer different view options with joystick-based control that allow unexperienced users to familiarize themselves with each function, while expert users can simultaneously use all functions.
- **Surrounding Recording**: A recording module is introduced into the system to offer the ability to document the robot’s daily activities, even if the user is not online. This guarantees robotic surveillance and the option of documentation.
- **Indoor–Outdoor Capability:** The proposed robot framework utilizes two wheels with heavy-duty hardware capability, supported by our system, which can control all moving wheels in any environment.

Ultimately, the current MRP systems need further development. AI technologies should be utilized to help more people with disabilities, the user experience should be considered when developing the system, the social aspect should be considered for distance communication, and the option for free accessibility is an important factor to introduce.

## 3. Elicitation Study

To develop the most efficient MRP system, it is important to locally understand the users’ expectations and the current state of MRP. Thus, an online questionnaire was distributed to possible users from our local community. The sample was randomly selected from Saudi Arabia and included 53 participants, with 66.7% being female and 33.3% being male. The sample contained people from different age groups: 18.8% were younger than 21 years old, 51% were between 21–30 years old, and 31.6% were older than 31 years old. Regarding the sample disabilities, 31.5% of the participants had a speech disability and 37% had a mobility disability at some point in their life.

As illustrated in Table 2, different questions were asked throughout the questionnaire. Concerning the accessibility of MRP robots, it was found that although approximately 39% had seen a telepresence robot, more than 61% have never used one. This was attributed to the fact that robots in Saudi Arabia are not very accessible to the public, although more expensive electronic wheelchairs and other tools are easily accessible. This issue highlights the need for more local development in that area to make them more accessible for public use. Moreover, regarding their preference for attending meetings online, the majority (67%) preferred to attend meetings using a robot that they could control through their phone. In terms of how the users would prefer for the robot to attend, most of the sample (83%) would prefer to have the robot go to the remote destination on their behalf instead of accompanying them.

Regarding TTS, most of the participants (81%) preferred to have the robot talk on their behalf. Interestingly, both people with a mobility disability and those with a speech disability agreed that they would prefer to have the robot talk on their behalf, possibly because they may be shy and have difficulty with direct communication. Finally, regarding the audio–video streaming, most of the sample preferred to have the robot stream its surroundings and give the choice to stream the user’s surroundings; 85% agreed on the need to show the robot’s surroundings, and 76% wanted to stream their own surroundings.

As a result of the elicitation study discussed so far, the proposed system should perform the following features.

- **Full Control:** The user should be able to fully control the body and head of the robot (upward, backward, left side, right side) to have a complete view of its surroundings and move it in any environment.
- **Audio–Video Communication:** The system should facilitate audio–video streaming from the side of the robot and the user should have complete control (start/stop/mute).
- **Text-to-Speech Communication:** The system should allow the robot to understand the text written by the user and speak it when commanded to do so.
- **Emotion Communication:** The system should allow the robot to show different emotions using its audio/screen capabilities based on the user commands.
- **Recording:** The system should allow the user to command the robot to record its activity and store it for later inspection.

In addition to the functional requirements, it is important to address the qualification criteria that will be used to measure the acceptance of the proposed system by the users.

- **Performance:** As confirmed by Shiwa et al. [22], since it is impossible to have an immediate system response time (SRT) of zero, the robot in our system should respond to the user commands within 1–2 s.
- **Usability:** The system usability scale (SUS) [26] should be measured during the testing of the system, and the score should be above 90 to be considered highly usable.
- **Availability:** The robot should be available at least during working hours, in addition to transportation breaks, with an estimated time of 10 h a day.

This system will facilitate physical interactions for people with special needs, and it can also be used by people to conduct in-person meetings, deliver something without physically meeting the other people, or follow-up with children who study away from home while the parents are working.

## 4. Proposed MRP System

**Figure 2:**
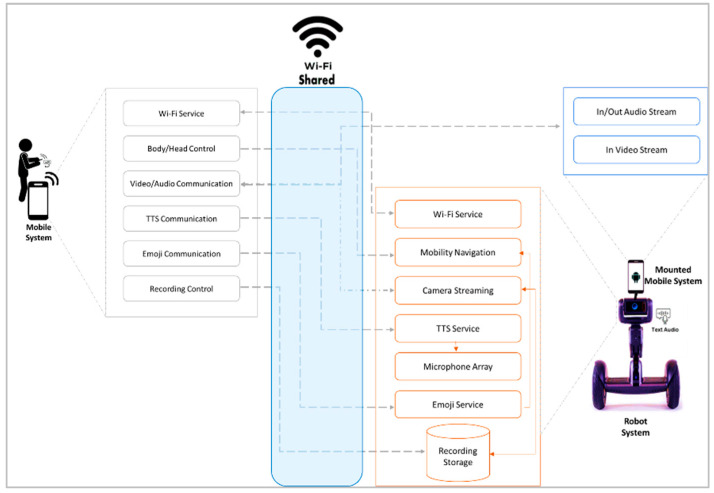
Framework of the proposed MRP system.

Based on comparative and elicitation studies, a new publicly accessible and user-centered design of an MRP system should be developed using a simple and portable control device such as a mobile phone. Thus, this section introduces the details of a new MRP system that was developed for people with a mobility or speech disability. To the authors’ knowledge, the proposed system is the first MRP system that supports speech impairments and introduces emotion components to its communication function. Starting with the system framework in Figure 2, the proposed MRP system includes three main components: the robot, the user’s mobile device, and the mounted mobile application. The robot application is used to control the telepresence robot; the mobile application is used to control the telepresence robot from a distance using Wi-Fi; the mounted mobile application is used as an additional sensor that has its own application to provide 2-way communication (audio–video) between the user and the robot (this sensor was added because the robot does not include audio–video sensors on both sides).

On the robot side, the modules are more related to controlling the robot motors for navigation and head movements, the speaker for TTS, and the camera for video streaming and recordings that are locally stored. Meanwhile, the mounted mobile modules are related to two-way audio streaming and the video from the pilot user. There is a very simple interface that allows local users to see and hear the pilot user from a distance. Finally, regarding the pilot user mobile device, its module allows the pilot user to connect to the robot and send his or her commands to the robot. These commands allow the user to control the robot’s motion and emotions using the emojis, camera, recordings, speaker, and TTS engine. It also sends commands to the mounted mobile device to start or stop streaming.

The interface of this system is easy to use to help users with mobility or speech impairments interact with others using two-way data streaming. AI technologies such as TTS are utilized to allow people to efficiently use the system. An emotion component is added to the system to improve social communication, which is very important because people with disabilities may find it difficult to communicate with other people. The system also supports video–audio streaming with the ability to record such streams on command. To understand each component, it is important to dive into the systems from three main levels: hardware, functional, and implementation.

### 4.1. Hardware Level

**Figure 3:**
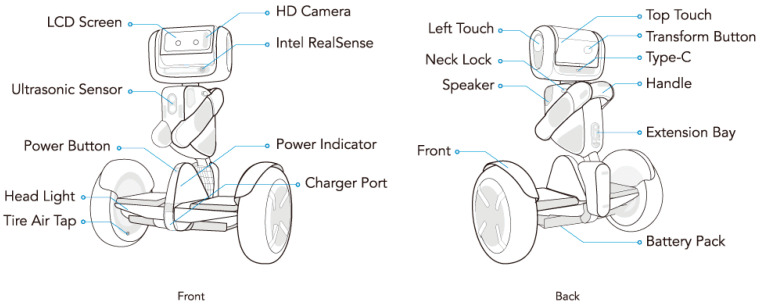
Loomo robot components [28].

In this paper, the robot controlled by the user (pilot user) using a simple mobile screen to interact with the other users (local users) at the remote location is called the Loomo robot [27]. The Loomo robot, which is shown in Figure 3, is a robot with an “*open platform that allows engineers and designers to build robot applications and accessories*” [27].

The robot includes different sensors and facilitates perceptive features that can be utilized for the proposed system [29].

- **Mobility:** The Loomo robot is designed with two self-balancing wheels to cover a distance of 30 km with only one charge, and can continuously work for 8–10 h.
- **Vision:** The Loomo robot is supported by various vision sensors such as Intel’s ReaSense module, which takes RGB-depth pictures at 30 frames per second, and an HD camera, which enables high-definition 1080P captures, accurate depth sensing, and fluid motion tracking. The cameras on the board can be viewed from multiple angles via the robot’s head.
- **Speech:** The Loomo robot facilitates perceptive features such as voice recognition and TTS communication; thus, it includes a microphone array of five microphones.
- **Navigation:** The Loomo robot head’s include a touch screen that allows users to manually control the robot; the IMUs in the robot body and head measure the robot’s poses and head orientations, while hall sensors are included in the wheel motors to give consistent odometry data; thus, the users can control the wheel alignment according to their needs.
- **Computation:** The Loomo robot includes a powerful 4-core Intel Atom Z8750 processor with a maximum frequency of 2.4 GHz, which enables the robot to run compute-intensive perception algorithms.
- **Hardware Extension Bay:** The Loomo robot offers users direct access to a USB 2.0 connector and a 24-volt power supply to power external devices.

### 4.2. Functional Level

**Figure 4:**
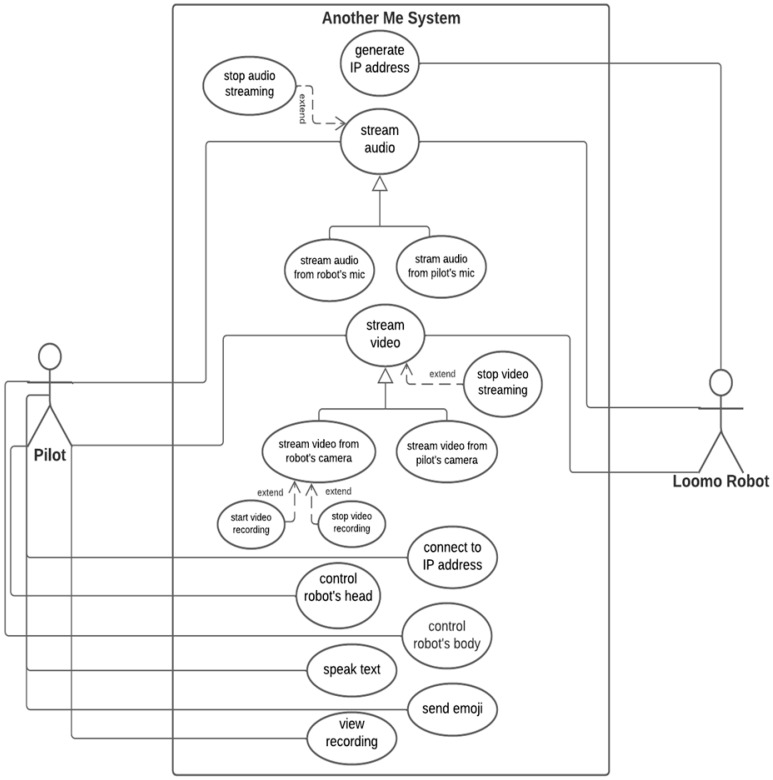
System components of the proposed MRP system.

As illustrated in Figure 4, the proposed system has different components, each of which facilitates a certain functionality. On the user’s side, the proposed system allows the pilot user to control the robot from a distance through the same Wi-Fi connection and allows the following actions to be performed:

- Control the body movement of the robot (forward, backward, and side movements);
- Control the head movement of the robot (up, down, and side movements);
- Send a text message to the robot to speak on behalf of the user;
- Send an emoji to the robot to show certain emotions through the robot’s head and screen;
- Display the robot’s camera stream on the user’s mobile application;
- Share the audio of the local environment on the user’s mobile application;
- Share the audio–video streaming from the pilot user on the mounted mobile device to communicate with local users;
- Record the local environment via the robot’s camera and store it on the robot’s memory to be viewed any time.

On the robot side, the robot at a remote distance will move and speak to the local users according to the pilot user’s commands. Thus, the robot and mounted mobile device will allow the robot to:

- Receive audio from the user’s mobile device and share it with local users;
- Receive video from the user’s mobile device and display it to local users;
- Speak the text sent by the pilot user;
- Show a certain emotion based on a received emoji command, whereby the robot will move its head and display on its screen an emotion that represents the requested emoji.

### 4.3. Implementation Level

**Figure 5:**
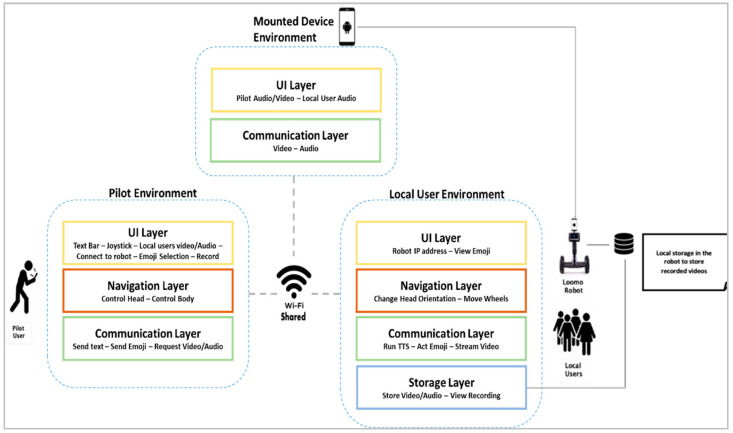
Architecture of the proposed MRP system.

The system explained thus far includes different architectural components, each of which works at a different layer in the proposed framework. Thus, it is important to understand the details of each layer, including the technical details, and to highlight the benefit of adopting such a component into the proposed system. In particular, three main applications are implemented. For the pilot user’s mobile device, an Android mobile application is developed for download on the pilot user’s mobile device. For the mounted mobile device, another Android mobile application is developed and downloaded on the mounted mobile device to be used with the camera and mic sensors. Finally, for the robot, an Android-based robot application is developed to allow the pilot user to control a robot from a distance and interact with the local users at a remote location. Therefore, the system architecture in Figure 5 shows that each component has its own UI, navigation, communication, and storage modules.

#### 4.3.1. User Interface (UI) Layer

**Figure 6:**
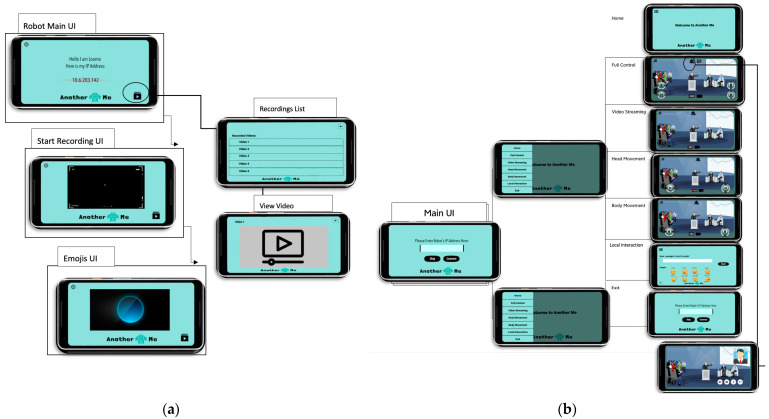
Components of the UI Layer. (**a**) Robot UI; (**b**) Mobile UI.

To provide a flexible and simple system that can be used by the user at any place and time, the platform used to build the system is the official Integrated Development Environment (IDE) Android Studio (Version 3.0.1). Through the mobile UI in Figure 6, the system offers a simple interface using a hamburger navigation bar while ensuring the following functions:

- Reduces confusion of the motion direction by complementing the joystick with arrows and identifying each stick with H and B letters to refer to head and body joysticks;
- Offers error prevention and simple error-handling techniques through validation and confirmation messages;
- Offers a test interface without connection to allow the users to familiarize themselves with the system before starting to use it;
- Offers an adaptable interface by designing different view options that allow unexperienced users to familiarize themselves with each function, while expert users can use all functions at once;
- Offers a consistent interface by using contrasting colors in the system’s background, buttons, and joysticks for a better user experience and to avoid distraction.

During the test and development cycle, decisions were made over certain UI modules to be removed or altered, as explained below.

- The TTS and emojis functions were implemented on separate interfaces at the beginning. However, after further discussion and consideration of the best UI experience, it was decided to merge them into one page. To confirm this decision, possible users were asked during the user acceptance test (as discussed in the following section) if they preferred to have these two functions on one page, and most of the sample strongly agreed. The reason is that most users would like to use the emoji functionality while sending the spoken text to the robot.
- The start–stop recordings option was also implemented on the robot UI in addition to the pilot UI. However, it was decided to remove it from the robot side due to security concerns; for example, outside users could have the ability to stop a recording that was initiated by the pilot user.
- All features of the audio–video communication system, such as mute–unmute, start–stop, or flip camera, were implemented on the mounted mobile in addition to the pilot interface. However, after further discussion and benchmarking of other telepresence and telecommunication tools, it was decided to remove it because local users might overtake the robot, which would not allow the pilot user to control it. To confirm this decision, possible users were asked during the user acceptance test (as discussed in the following section) whether they preferred to have full control over the robot communication, and more than half of the users preferred to have full control on their side.

#### 4.3.2. Navigation Layer

**Figure 7:**
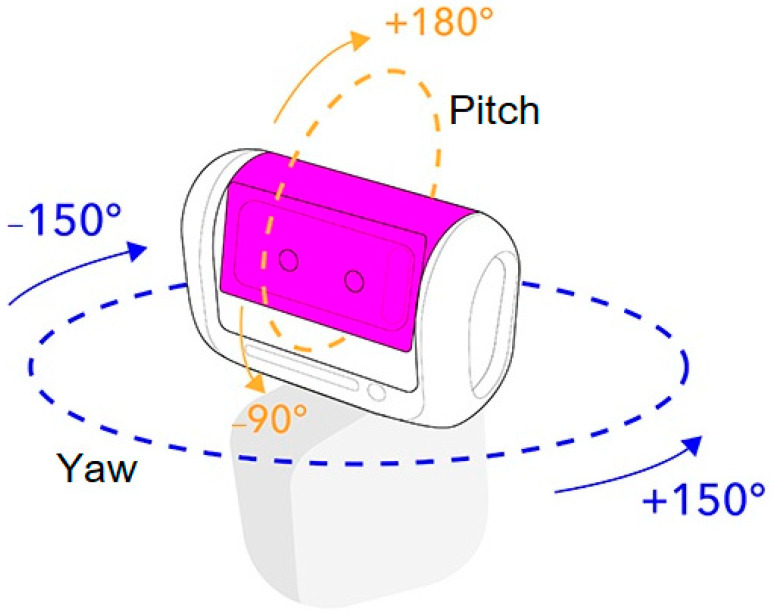
Head angle ranges in the navigation layer [28].

To allow the user to fully control the robot’s head and body, it was important to understand the concept behind the Loomo robot navigation system. Starting with the head navigation system illustrated in Figure 7, the robot has a screen with two dimensions (called pitch and yaw) to enable control of the robot’s head direction. The pitch dimension can be controlled to move the robot’s head up and down, while the yaw dimension can be controlled to move the robot’s head right and left.

The implemented SDK provides different head modes, including the SMOOTH_TRACKING_MODE and LOCK_MODE, as explained below. To change the position of the robot’s head, the LOCK_MODE was implemented to enable the robot’s head to move with a specific velocity and to control the head orientation by setting the head rotation velocity.

- SMOOTH_TRACKING_MODE: The head’s pitch axis is stable, while the head’s yaw axis can rotate following the base. It is controlled by setting the Loomo base angle to be identical to the head movement angle. For example, if Loomo’s base turns to the left by 45 degrees, the head will also turn to the left by 45 degrees.
- LOCK_MODE: This mode enables the robot’s head to move with a specific velocity. The head’s pitch axis is stable, while the yaw axis points in a certain direction in the world coordinate system. Hence, it controls the head orientation by setting the head rotation velocity.

**Figure 8:**
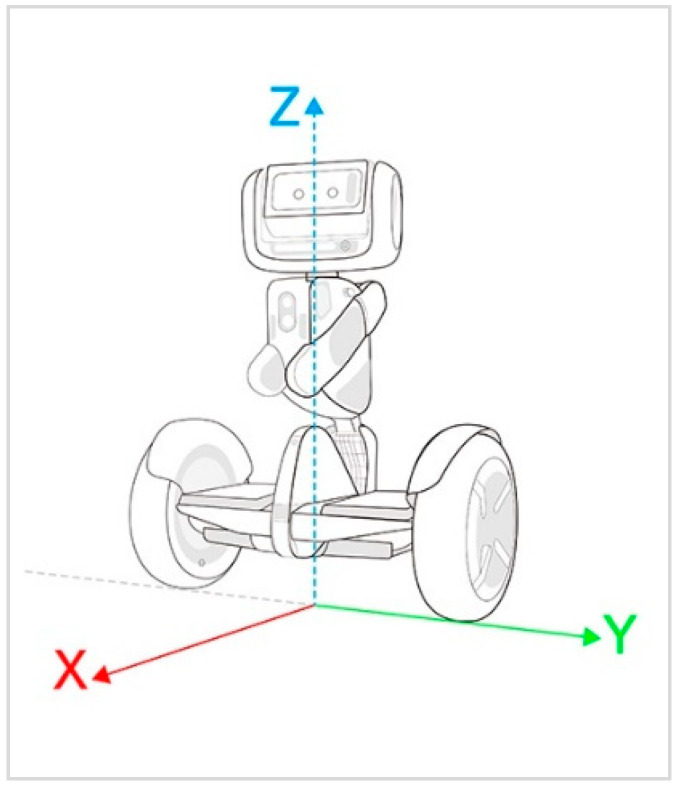
Robot body reference frame at the navigation layer: X and Y represent the linear velocity and Z represents the angular velocity [28].

Regarding the body motion of the robot, the self-balancing two-wheel structure helps navigation through indoor and outdoor environments. As shown in Figure 8, the robot can navigate and move from one location to another using a group of SDK functions to work together. For navigation, there is a need to specify a value for each axis, X, Y, and Z, where X and Y represent the linear velocity and Z represents the angular velocity. The *X* axis enables the robot to move forward and backward using positive and negative values, respectively. Meanwhile, the Y axis controls the robot’s body motion from right to left, and the *Z* axis controls the robot’s angle to move in the counterclockwise direction (positive values) or clockwise direction (negative values).

The implemented SDK enables one to control the motions using different modes, namely CONTROL_MODE_RAW, CONTROL_MODE_NAVIGATION, and CONTROL_MODE_FOLLOW_TARGET, as explained below. For simple navigation control, it was decided to implement the CONTROL_MODE_RAW mode, since it can allow the user to move the robot body by a simple click of a button.

- CONTROL_MODE_RAW: This mode enables the system to manipulate the robot’s speed by changing the velocities and passing a number to set the linear and angular velocities.
- CONTROL_MODE_NAVIGATION: This mode enables the robot to navigate in a certain path by identifying points to navigate over a predefined map. It adds checkpoints that identify X, Y, and Z values to move the robot from its current position to a new point.
- CONTROL_MODE_FOLLOW_TARGET: This mode enables a “follow me” scenario. The robot will try to move to a new point by receiving a distance and a direction relative to itself to track a person’s steps.

#### 4.3.3. Communication Layer

In the communication layer, the user can communicate with others in three ways: video–audio streaming, TTS, or emoji communication. Regarding video–audio streaming, the system is used to collect the images captured by the robot’s Intel RealSense camera in the Bitmap format. Then, these data are compressed into the JPEG format and transferred as an array of bytes measuring 1 MB to the mobile application to be transferred back at the pilot user side and viewed as video files. Meanwhile, to stream the audio, since audio–video files are streamed from different sensors, the system will first check if the user gave access permission to the audio. If yes, the system will enable the audio transfer directly through the network because this option is activated through the mounted mobile speaker instead of the Loomo robot sensors.

**Figure 9:**
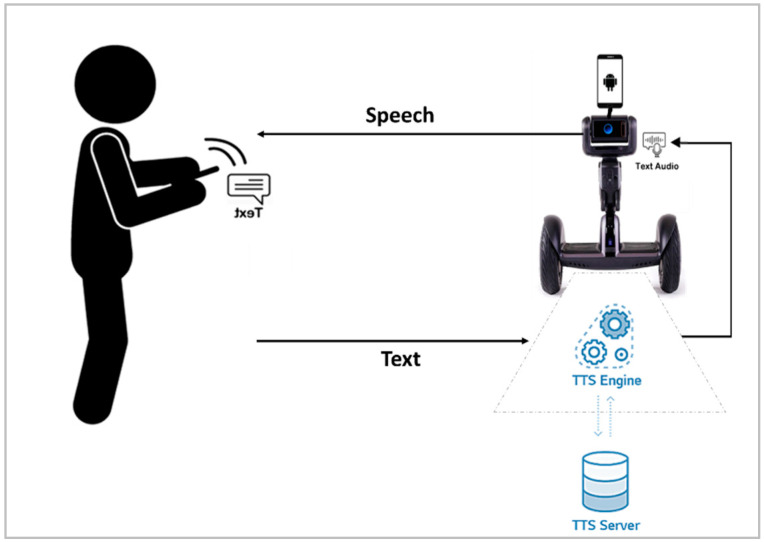
TTS communication in the communication layer.

Regarding TTS communication, to allow the robot to speak the text sent by the pilot user, it is important to use a TTS engine. Loomo provides a TTS engine, which gives the robot the ability to read text aloud. The TTS engine converts written text to a phonemic representation and converts the phonemic representation to waveforms that can be output as sound. As illustrated in Figure 9, the TTS engine used from Loomo SDK services converts written text to a phonemic representation and subsequently converts the phonemic representation to waveforms that can be output as sound. In the developed system, the robot speaker converts the text to speech after binding it to the TTS service to speak through the speaker service.

**Figure 10:**
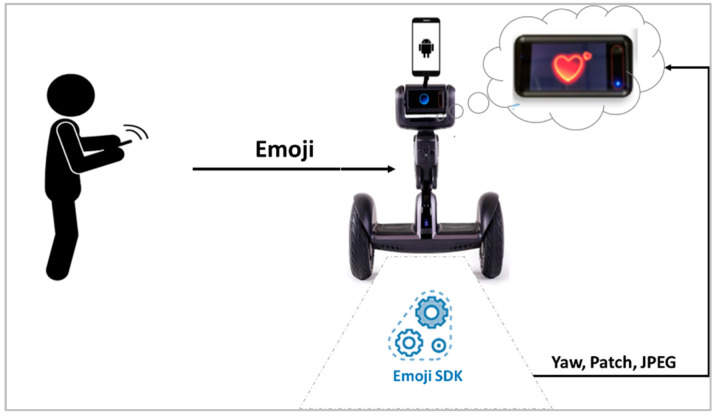
Emoji communication in the communication layer.

Finally, regarding the emoji communication, the developed application uses an Emoji SDK. As illustrated in Figure 10, this SDK allows the robot to coordinate and play emotions, sounds, and actions on its screen. Using Loomo’s default expression library and by changing the yaw and pitch velocities, the robot can “look down”, “look up”, “look around”, show “curiosity”, “show happiness”, or “show sadness”, as well as other types of emotions.

#### 4.3.4. Stage Layer

In the developed system, the user can directly start the recording from the robot side by simply selecting the recording button from the mobile UI. When the user starts the recording command, the robot camera will be activated and permission will be given to the robot application side to start recording the video stream. When the user stops the recording, the video will be locally stored into the robot’s memory to be viewed later by the user using the robot UI. To view the recorded video, a new library called Vitamio was used to create a view interface in the developed system and to help the user view the recorded video through the robot screen.

## 5. Experimental Study

To measure the validity of the proposed system, a user acceptance test (UAT) was conducted. The Standing Committee for Scientific Research Ethics approval was granted to conduct the experiment on people with and without disabilities (application # 21-763), and different types of people were invited to test the system.

### 5.1. Experimental Setup

**Figure 11:**
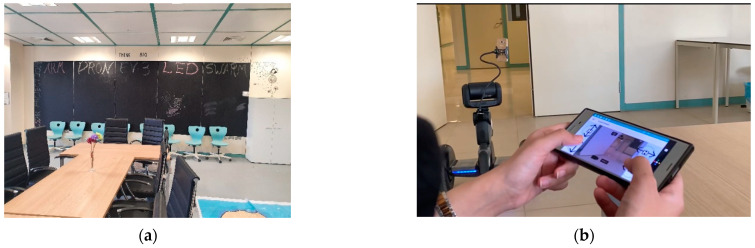
Experimental Setup. (**a**) Pilot user room; (**b**) Controlling the robot to go to the meeting room.

To assess the system, the experiment occurred at King Saud University campus, and the pilot user (participant) was placed in a CS2R center (room 25), as illustrated in Figure 11a. It was decided to imitate a work environment where the pilot user uses the robot in the CS2R center (room 25) to attend a meeting in another place with two of the team members. Thus, a meeting was held in another room with the two team members. Then, the user started the system to control the robot, as illustrated in Figure 11b, and navigated the floor to go to the meeting room. Thereafter, the participant was given full control to try the system. All participants were invited to the test center at different times and did not meet each other during the tests, so that they could formulate their own opinion.

Thirty participants were invited to test the system. They were invited in two phases: foreseeing and validating. The foreseeing phase was conducted in the middle of the development to validate existing developments to foresee future improvements. This test was conducted with 10 participants, two of whom had a mobility disability. Meanwhile, the validation phase was conducted on the final product to measure how well it articulated the users’ requirements. This test was conducted with 20 participants, four of whom had a mobility or speech disability. Participants with disabilities were invited from the “Harakia” Disability Organization (https://harakia.org.sa/ 1 August 2022) and the “KSU” Disability Center (https://womencampus.ksu.edu.sa/ar/node/281 1 August 2022).

**Figure 12:**
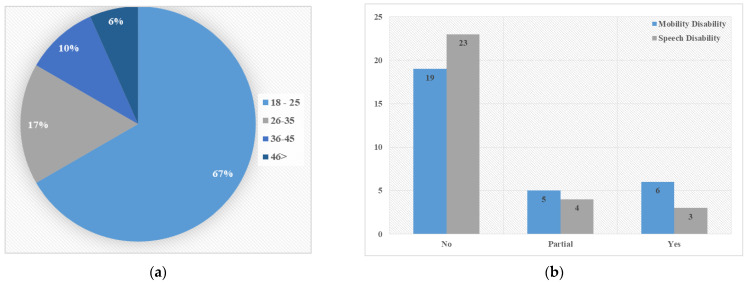
Participant demographics. (**a**) Age distribution; (**b**) Disability distribution.

Regarding the participants’ demographics, all were female due to the limitation of male access to the all-female campus. As illustrated in Figure 12, the participants’ age range was 1–45 years; 10% of the sample had a speech disability, 13% of the sample had temporarily experienced a speech impairment in the past, 20% of the sample had a mobility disability, and 17% had temporarily experienced a mobility disability in the past.

### 5.2. Experimental Results

After completing the test sessions, each candidate was given a survey to complete and subsequently separately interviewed to provide detailed feedback about the system. In the survey, the answers were rated based on five scales (excellent, very good, good, poor, and very poor). The questions were grouped into three groups: mobile system review, robot hardware review, and robot system review. In general, from all collected results, no participant rated any criteria as poor or very poor, which indicated a high satisfaction rate.

**Figure 13:**
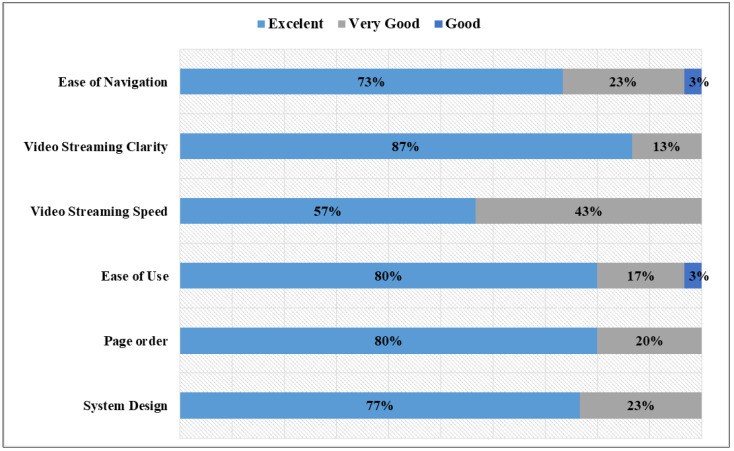
Feedback on mobile system components.

Starting with the questions related to the mobile system, which are illustrated in Figure 13, the participants found the system to be easy to navigate and the design and interface to be user-friendly (80% of the participants rated these criteria as excellent). Interestingly, almost half of the participants rated the video streaming speed as very good, while the other criteria were mostly rated excellent. A further investigation showed that the streaming speed was affected by the network stability. In particular, when the test was conducted early in the morning, many classes were taking place in the building and many people were using the network, which affected the network stability; thus, the speed of video streaming between the robot and mobile device was low. Thus, it was recommended to access a private network when using the system to utilize the shared network for the pilot user.

**Figure 14:**
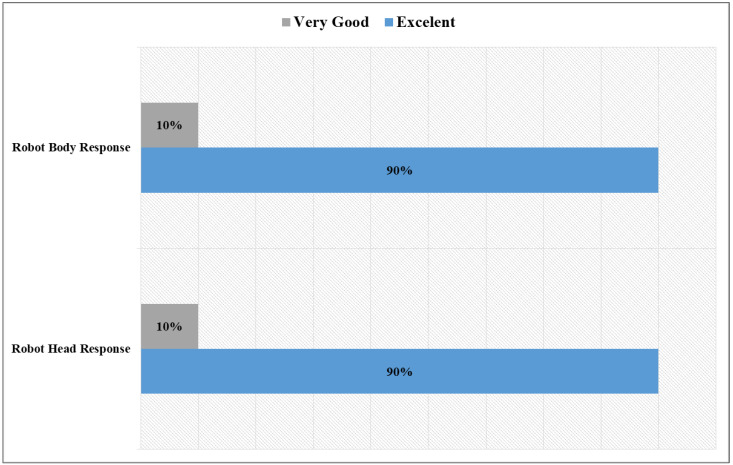
Feedback on robot hardware components.

In addition to the mobile system, it was important to test the robot’s hardware response to the system, especially in the first phase of the test. As shown in Figure 14, only one participant rated its responsiveness as very good, while the remainder rated it as excellent. Thus, the navigation command and communication systems were not highly affected by the network as in the video streaming, which is understandable because each navigation command is only an 8-byte text message.

**Figure 15:**
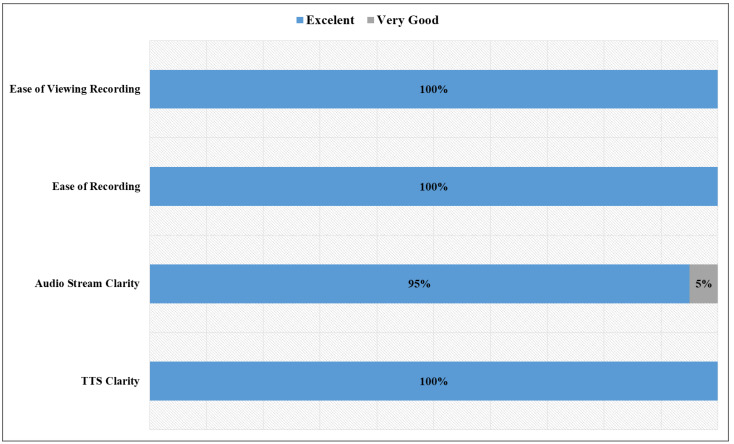
Feedback on the robot system components.

Finally, regarding the system implemented in the robot, the participants were asked to rate sending a text to the robot, showing emojis, joining others in a video call, and recording. As shown in Figure 15, all participants were very satisfied with using the robot. Only one participant rated the audio streaming clarity as very good. This result suggests that the users had fun working with the robot and confirmed the literature findings on how to help users better engage with robots.

After finishing the survey, each participant was interviewed and asked about their experience with the system. In the interview, the participants were asked about some of the design choices that we considered in the system. For example, they were asked whether they preferred having the option of additional pages that separate navigation from streaming, and 75% of the sample answered yes. In particular, one participant who had a mobility disability also had difficulty in controlling her left hand, so she emphasized that having this option allowed her to better control the robot, since she could not control all joysticks on the main page. Moreover, the TTS and emoji components were merge into one page to allow the users to send their emotions with the text that the robot says; when the participants were asked about this merge, 70% of the sample preferred it this way. Additionally, allowing only the pilot users to control the audio–video was preferred by more than half of the sample.

During the experiment, the participants stated that they were excited about the idea of the system and eager to try it out; one participant requested to use the system after the test and walk around to say hello to students on campus. After this experience, the user was very happy and liked how others engaged with the robot and how they interacted with her through it. The users found the system helpful, and they were interested in using it in the future. At the end of the experiment, the users provided suggestions to increase the value of the application, such as letting the user select the speed of the robot movement, control the robot movement by voice, and support the Arabic language.

Finally, regarding the qualification criteria and based on the collected results, it was possible to evaluate each criteria of the system as follows.

- Performance: During the experimental study, the SRT time was measured with each participant command. It was confirmed that the robot reacted based on the user commands in less than two seconds every time.
- Usability: To measure the usability of the system, the SUS score [21] was computed by distributing a 10-item questionnaire to the participants and calculating the SUS score. The resulting score was 95.87, which indicated that the acceptance rate was in the 99% percentile rank and that the system was highly usable. In conclusion, based on all discussed results, the system evaluation was successful. The participants were very excited and looked forward to seeing the application ready and available for people to use.
- Availability: During the experimental study, the robot was left on and working for more than 10 h to confirm that the availability measure was satisfied.

## 6. Conclusions

People with special needs often face difficulties when communicating with other people because of logistical or physical challenges. Communication and socialization can be a struggle, and due to their frustration, many people with disabilities avoid social events or face-to-face meetings. Thus, in this paper we proposed an MRP system that helps people with mobility or speech disabilities overcome the difficulties that they face in communicating and normally engaging with other members of society. The developed system allows people with a mobility disability to freely navigate in different environments using a simple and user-friendly mobile application, facilitates communication for people with speech impairments using TTS, and manages space and time for the user through audio–video streaming and recording functions from the remote space. This system will facilitate physical interactions for people with special needs and can be used by people to conduct in-person meetings, deliver items without physically meeting other people, or even follow up with children who study away from home while the parents are working. This application is of particular importance due to the current recommendations to reduce physical interactions during the current COVID-19 pandemic. The system evaluation showed that the system successfully satisfied the user needs, and participants found the system helpful and were satisfied with its application. In the future, the system will be improved by supporting the Arabic language for TTS, and the usability of the robot’s joysticks will be improved to allow one joystick to fully control the robot’s movement.

## Figures and Tables

**Table 1:** Features of the current MRP systems.

Robot	User	Accessibility	Video	Audio	TTS	Emoji	Recording	Controller
Kubi [18]	Students	Commercial	2 Ways	2 Ways	No	No	No	Keyboard
Robovie- R3 [20]	Students	Commercial	2 Ways	2 Ways	No	No	No	Data Glove
Pioneer 3AT [21]	Visually Impaired	Commercial	1 Ways	1 Ways	No	No	No	Keyboard Screen touch
Pi [3]	Mobile Impairment	Research	2 Ways	2 Ways	No	No	No	Not Clear
QB [18]	Employees	Commercial	2 Ways	2 Ways	No	No	No	Keyboard
TeleMe [22]	Employees	Commercial	2 Ways	2 Ways	No	No	No	Butten Control Panel
Beams [23]	Academic	Commercial	2 Ways	2 Ways	No	No	No	Keyboard Mouse Drag on Screen
Double [24]	Students	Commercial	2 Ways	2 Ways	No	No	No	Click to Move Screen
Vgo [19]	Students	Commercial	2 Ways	2 Ways	No	No	No	Mouse
Marvin [25]	Elderly	Research	2 Ways	2 Ways	No	No	No	Voice Screen

**Table 2:** Elicitation study questionnaire.

Questions	Answers
Have you used or seen a telepresence robot before? Yes Maybe No	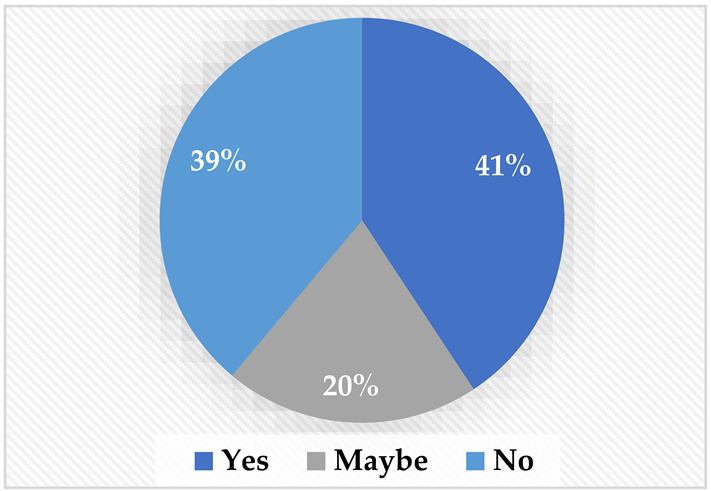
If you want to connect with a group of people, what would you prefer to be the means of communicating with them? Call via phone or video (such as Zoom and Teams). Using a remote robot that moves from one place to another through an app on your phone. Other.	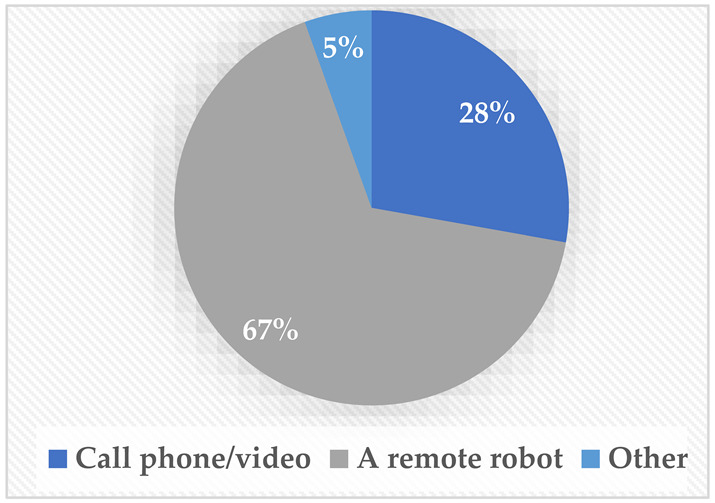
If there was a robot that you can control remotely, would you prefer: The robot to accompany you and talk based on your orders. To send the robot on your behalf and stream what is happening around it while you control It.	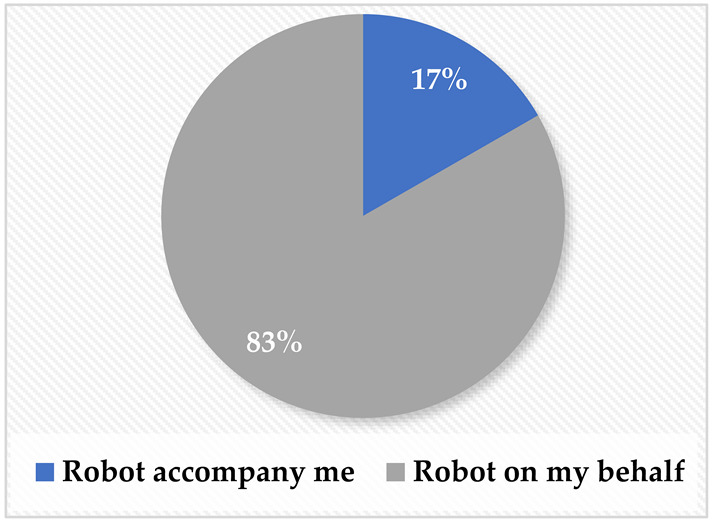
Would you rather have a feature from which you can make the robot translate your text message into words spoken by the robot for you? Yes. Sometimes. No.	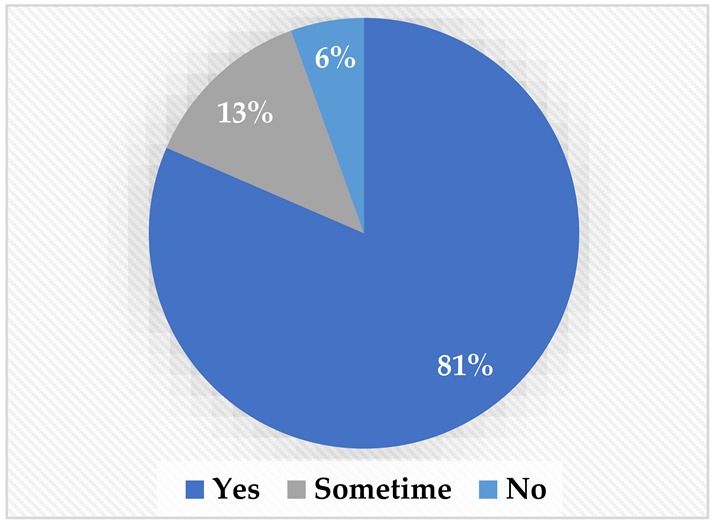
Would you rather see and hear the people in front of the robot? Yes. Sometimes. No.	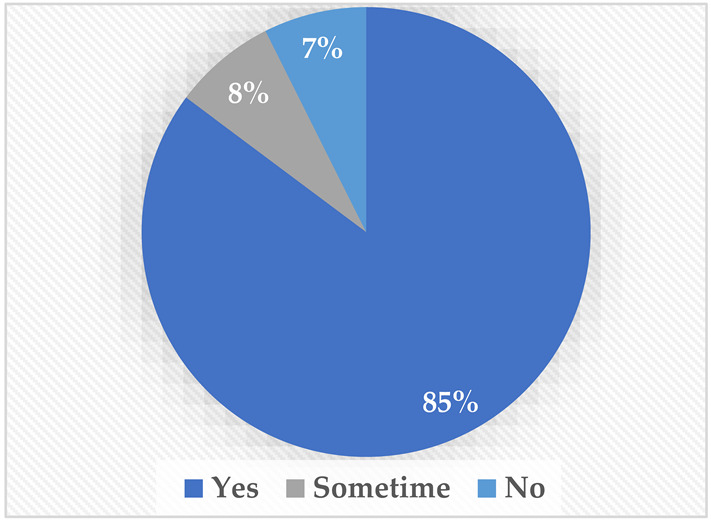
Would you rather have a feature where you can stream your voice and image to the people in front of the robot? Yes. Sometimes. No.	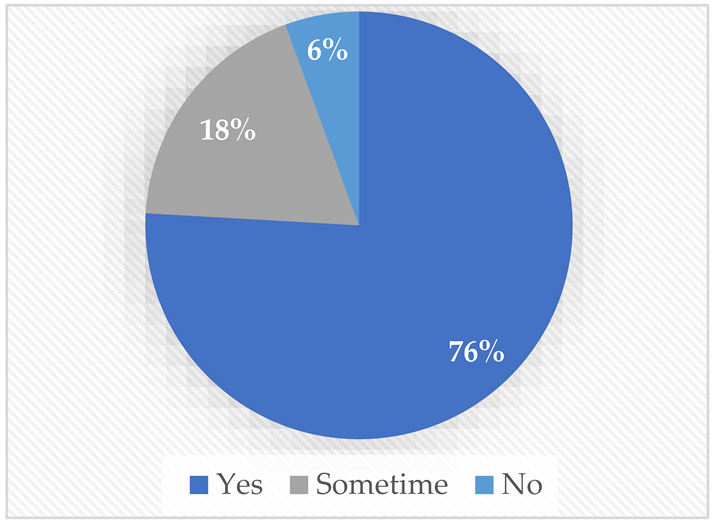

## Data Availability

Not applicable.
